# Mixed-phase weak anion-exchange/reversed-phase LC–MS/MS for analysis of nucleotide sugars in human fibroblasts

**DOI:** 10.1007/s00216-024-05313-w

**Published:** 2024-04-27

**Authors:** Moritz Rahm, Hanneke Kwast, Hans J. C. T. Wessels, Marek J. Noga, Dirk J. Lefeber

**Affiliations:** 1 Department of Neurology, Donders Institute for Brain, Cognition, and Behavior, Radboud University Medical Center, Geert Grooteplein 10, 6525 GA Nijmegen, The Netherlands; 2 Translational Metabolic Laboratory (TML), Department of Human Genetics, Radboud University Medical Center, Geert Groote Plein Zuid 10, 6525 GA Nijmegen, The Netherlands; 3 Laboratory of Clinical Genetics, Inborn Errors of Metabolism, Maastricht University Medical Center, Maastricht, The Netherlands

**Keywords:** LC–MS/MS, Mixed-mode chromatography, Nucleotide sugars, Sugar metabolism, Congenital disorders of glycosylation

## Abstract

Nucleotide sugars (NS) fulfil important roles in all living organisms and in humans, related defects result in severe clinical syndromes. NS can be seen as the “activated” sugars used for biosynthesis of a wide range of glycoconjugates and serve as substrates themselves for the synthesis of other nucleotide sugars. NS analysis is complicated by the presence of multiple stereoisomers without diagnostic transition ions, therefore requiring separation by liquid chromatography. In this paper, we explored weak anion-exchange/reversed-phase chromatography on a hybrid column for the separation of 17 nucleotide sugars that can occur in humans. A robust and reproducible method was established with intra- and inter-day coefficients of variation below 10% and a linear range spanning three orders of magnitude. Application to patient fibroblasts with genetic defects in mannose-1-phosphate guanylyltransferase beta, CDP-l-ribitol pyrophosphorylase A, and UDP-*N*-acetylglucosamine 2-epimerase/*N*-acetylmannosamine kinase showed abnormal levels of guanosine-5′-diphosphate-α-d-mannose (GDP-Man), cytidine-5′-diphosphate-l-ribitol (CDP-ribitol), and cytidine-5′-monophosphate-*N*-acetyl-β-d-neuraminic acid (CMP-Neu5Ac), respectively, in consonance with expectations based on the diagnosis. In conclusion, a novel, semi-quantitative method was established for the analysis of nucleotide sugars that can be applied to diagnose several genetic glycosylation disorders in fibroblasts and beyond.

**Supplementary Information:**

The online version contains supplementary material available at 10.1007/s00216-024-05313-w.

## Introduction

Nucleotide sugars (NS) play crucial roles in cellular metabolism providing activated sugars for glycosylation of proteins and lipids, glycogenesis, and as intermediates in metabolic pathways like the Leloir or the uronic acid pathway. Nucleotide sugars are synthesised in mammals through de novo synthesis or via salvage pathways. They can also be synthesised from other nucleotide sugars through direct isomerization, such as uridine-5′-diphosphate-α-d-glucose (UDP-Glc) to uridine-5′-diphosphate-α-d-galactose (UDP-Gal), or via a series of enzymatic reactions, such as guanosine-5′-diphosphate-β-l-fucose (GDP-Fuc) from GDP-Man [1]. Many enzymes involved in sugar metabolism are known, although novel mechanisms and nucleotide sugars are still being identified, such as uridine-5′-diphosphate-α-d-mannose (UDP-Man) and low abundance uridine-5′-diphosphate-β-l-arabinose (UDP-Ara) in human cells [2, 3].

Alterations in nucleotide sugar profiles have been reported in human disease, including cancer [4, 5]. A specific group of monogenic diseases, congenital disorders of glycosylation (CDG), can affect sugar metabolism causing abnormal levels of specific nucleotide sugars [6–8]. CDGs with abnormal N-glycosylation were classically split into two types, where in type 1 CDG the attachment of N-glycans to nascent proteins is affected in the endoplasmic reticulum, while in type 2 CDG the processing of N-glycans is deficient. Defects in sugar metabolism can either result in type 1 CDG (such as PMM2-CDG) or in type 2 CDG (such as SLC35A2-CDG) [9]. This classification is based on the glycoprofiling of serum transferrin. In the last decade, a growing number of defects in sugar metabolism has been identified, mainly via next-generation sequencing, in which the N-glycosylation of transferrin is not affected, thereby complicating diagnosis. Examples include CRPPA-CDG and GMPPB-CDG [10].

As nucleotide sugars play an important role in cellular metabolism of every living organism and their role in human disease requires further mechanistic investigations, methods for their specific analysis are needed. Although there is a notable difference between NS profiles of plants, bacteria, and animals, the general molecular structure is very similar and composed of a nucleobase, a potentially modified ribose unit, and a monosaccharide. Most current analytical methods rely on chromatographic separation by hydrophilic interaction (HILIC), porous graphitic carbon (PGC), ion-pair (IP) reversed-phase, capillary electrophoresis, and anion-exchange (AX) chromatography. Mass spectrometry (MS) is a suitable detection and separation method often combined with liquid chromatography (LC). When applied in the form of tandem MS or MS/MS, it provides additional information through fragmentation making it useful for identification of nucleotide sugars. In addition, MS/MS provides higher sensitivity and selectivity as well as the possibility to differentiate between isotopically labelled and non-labelled compounds in case of dynamic tracing experiments using for example [U-^13^C]glucose [2, 11, 12]. MS also allows the use of stable isotope labelled internal standards to increase precision of quantification. However, not all NS are separable by these means of detection, in turn requiring an additional dimension of separation.

The range of separation methods as indicated above displays some issues that may prevent widespread utilization. HILIC allowed separation of isobaric nucleotide sugars UDP-Glc and UDP-Gal extracted from maize, although a gradient length of 40 min was required [13]. PGC is capable of separating up to 35 NS in different sample materials [14, 15]. However, PGC columns require thorough cleaning and regeneration to avoid inconsistencies throughout the column’s lifetime including shifts in retention time and disappearance of peaks [16] and samples prerequired solid phase extraction for matrix cleanup and concentration. Buscella et al. [17] made use of capillary electrophoresis for separation of nucleotide sugars. Conditioning the column with 1 M NaOH, distilled water, and a 200 mM boric acid (pH adjusted to 6 to 8) solution for 20 min each before starting measurements. Measurement cycle duration including capillary conditioning was reported to be above 70 min. While this method is the only one capable of separating the challenging analytes uridine-5′-diphosphate-*N*-acetyl-α-d-glucosamine (UDP-GlcNAc) and uridine-5′-diphosphate-*N*-acetyl-α-d-galactosamine (UDP-GalNAc) by more than a minute, this comes at the cost of throughput and compatibility with mass spectrometry [17].

Most reported methods make use of IP chromatography, which allows separation of nearly all nucleotide sugars but also requires extensive flushing and cleaning due to the adhesive nature of ion-pair reagents. Most ion-pair reagents often remain even after cleaning and therefore occasional use on multi-purpose mass spectrometers is not recommended [2, 18–20]. In view of the strong potential of ion-pairing methods, we explored the use of mixed-mode anion-exchange/reversed-phase liquid chromatography to separate NS by their affinity towards ion exchange moieties as well as by their hydrophobicity. This multi modal separation proved to provide higher selectivity for analytes containing multiple moieties with different chemical properties. We found that the similar retention mechanisms showed good performance to separate and analyse 17 human NS by LC–MS without the need for ion-pairing reagents.

## Materials and methods

### Materials

LC buffer components acetic acid (Supelco, purity ≥ 99.8%), ammonium acetate (Fluka, purity 99%), and acetonitrile (Biosolve, LC–MS grade) were purchased. Standards for nucleotide sugars (see Table S1) were obtained at the highest available purity from Sigma-Aldrich, while 2′deoxy-thymidine-5′-diphosphate-α-d-glucose (dTDP-Rha), uridine-5′-diphosphate-α-d-xylose (UDP-Xyl), and UDP-Ara were acquired through Biosynth. Milli-Q water was obtained from a Milli-Q purification unit (Milli-Q® Advantage A10, Merck Millipore) with resistance above 18 MΩ.

UDP-Man was obtained with courtesy of Dr. Kazuki Nakajima (Fujita Health University, JP).

### Patient fibroblasts

Fibroblasts of patients and controls were retrieved from the biobank of the Radboudumc expertise center for disorders of glycosylation and used in accordance with the Declaration of Helsinki revised in 2000 and ethical approval has been granted through the local “Commissie Mensgebonden Onderzoek” (CMO: 2020–6588). Cell lines were passaged at 70–80% confluence to a maximum of 25 passages and medium was refreshed twice a week. Patient and control fibroblasts were cultured in six-well plates containing 2 ml of medium. Each cell line was cultured in triplicate. Medium was composed of 10% dialysed FCS/DMEM (glucose free) to which 5 mM glucose was added. In total, 150,000 cells were seeded per well. The plates were placed in an incubator at 37 °C for 48 h when a target confluence between 80 and 90% was reached.

The pathological samples include muscular dystrophy-dystroglycanopathy (limb-girdle) type C (OMIM #616,052, also known as ISPD or CRPPA-CDG, referred to as patients A and B), GDP-mannose pyrophosphorylase B (OMIM *615,320, referred to as patients C and D), and sialuria, French type (OMIM #269,921, referred to as patient E). The fibroblasts from GMPPB and ISPD patients were cultured in the same experiment and therefore share the control cell line A. French type sialuria patient cell line was cultured in a second experiment with the corresponding control cell line B.

### Metabolite extraction and preparation

Cells were rinsed twice with 2 ml freshly prepared 75 mM ammonium carbonate (pH = 7.4) buffer and immediately frozen in liquid nitrogen. Frozen plates were stored at − 80 °C until extraction. The metabolite extraction procedure was performed once per well as described in van Scherpenzeel et al. yielding 100 µl of aqueous extract [2].

### LC–MS/MS conditions

LC separation was performed on an Atlantis Premier BEH C18 AX Column (2.1 × 150 mm 1.7 µm, Waters) weak anion-exchange/reversed-phase hybrid column. Mobile phase consisted of buffer A (Milli-Q water) and buffer B (50 mM ammonium acetate and 50 mM acetic acid in 5% acetonitrile and 95% Milli-Q water at pH 4.7). Column temperature was kept constant at 50 °C throughout the run. Flow rate was kept constant at 0.5 ml/min during the following time program (B in %): 0–3 min: 30%, 4–5 min: 33%, 10–12 min: 100%, 12.5–15 min: 30%. Column conditioning was performed at 30% buffer B, and 0.5 ml/min for 60 min prior to starting a series of measurements. Injected sample volume was 1 µl with a 20 µl sample loop and a flush-out factor of 5.

Mass spectrometry was done on a Sciex 6500 + QTRAP with an electrospray ionization source. Curtain gas was set to 35 psi, collision gas to “Medium”, and both ion source gas outlets to 50 psi. Ion source gas temperature was set to 650 °C and ion spray voltage to − 4500 V. Declustering potential, entrance potential, and collision cell exit potential were set to − 60 V, − 10 V, and − 12 V, respectively. Collision energy (CE) values were optimized in accordance to conventional practice measuring intensities in a standard mix (see Supplementary Table S3). As no standard was available for CDP-ribitol, fibroblast extract was used for transition optimization.

Of all patients and control samples, one extract each was measured as analytical triplicates in randomized order within one run. All transitions corresponding to the same precursor were added together. The peak area of all nucleotide sugars detected above the lower limit of detection (LOD) was summed to obtain the total peak area (TPA). Peak area of individual NS was then normalized against TPA and expressed as a fraction thereof.

Statistical analysis was performed in Graph Pad Prism version 9.5.1 treating each metabolite as an individual feature. Normality of data was tested with the Shapiro–Wilk test. Significance of difference in means between samples (95% confidence interval) was tested using one-way ANOVA with Bonferroni correction for GMPPB and ISPD and unpaired *t*-test for French type sialuria.

### Method validation

For method validation, the instrumental LOQ, linear range, intra- and inter-day variance, and carry-over were determined.

*LOD and LOQ* were determined from blank noise and a dilution series. The LOD and LOQ were defined as the concentrations that with 95% confidence produce a signal above the LOD (average + 3 SD blank noise) and LOQ (average + 3 SD blank noise) intensity cutoff, respectively. As intensity cutoff, the average and standard deviation of noise in 10 1-µl blank injections were determined. For the concentration-dependent LOD and LOQ, a dilution series of nucleotide sugar standard mix with concentrations 20 nM, 15 nM, 12 nM, 9 nM, 6 nM, 3 nM, and 1 nM were measured 10 times. For each replicate, a linear regression was fitted and the standard deviation of the slope and *y*-intercept was calculated. The 95% confidence intervals of slope and *y*-intercept and their lower confidence limit intercepts with the LOD and LOQ intensity cutoffs were calculated.

*In-source fragmentation* was evaluated by ramping the declustering potential in steps of 5 V from 0 V to − 150 V measuring a 1 µM standard mix in triplicate. The average values were normalized against the maximum intensity, plotted against the declustering potential and a generalized bell curve was fitted minimizing the sum of error. Maximum intensity plateau was defined as the highest 1% normalized intensities of the fitted curve. The range of corresponding declustering potential values was reported as optimum.

*Linear range* was determined by measuring of commercial standards (excl. UDP-Man and CDP-ribitol) that were split into four mixes of not isobaric or interfering standards (see Supplementary Table S4). The concentrations were 10 nM, 33 nM, 100 nM, 330 nM, 1 µM, 3.3 µM, 10 µM, and 33 µM. Standard mixes were measured in triplicate in order from lowest to highest concentration alternating between mixes to reduce interference between measurements. The Pearson correlation coefficient (*r*) was calculated for correlation between compound-dependent peak area and concentration. A linear regression model was calculated with applied weighting — x, 1/x, 1/x^2^, 1/x^0.5^ — and the best fit was determined by comparing the sum of relative error for each compound. Concentrations with a relative error of > 20% were determined as outside the linear range.

*Carry-over* was determined in accordance with IUPAC convention. Single standards were injected as high level at 33 µM and low level at 10 nM concentration.

*Intra- and inter-day* variance was determined from a standard mix of all commercial standards and UDP-Man at 200 nM concentration. Intra-day variance was determined from 10 replicates measured in 1 run, whereas inter-day variance measurements were performed on 10 consecutive days.

*Sample stability* was determined by measuring 1 µM standard mix in triplicate at timepoints ranging from 0 to 48 h after the samples were placed in the autosampler at + 4 °C. The peak areas of triplicates were averaged and expressed as relative difference compared to the 0 h timepoint without prior normalization. The standard deviation of all relative changes was calculated to determine the 95% confidence interval for all analytes.

*Matrix effect* was determined by matrix-matched standard calibration. First, nucleotide sugar profiles in two native fibroblast extracts A and B were measured and UDP-Ara, GDP-Glc, and ADP-Glc were chosen for matrix effect determination due to their absence in controls and retention times. Nine concentrations (15 nM, 30 nM, 60 nM, 120 nM, 240 nM, 480 nM, 960 nM, 1920 nM, 3840 nM) were chosen to cover the linear range. Ten-fold concentrated standard mixes were prepared in Milli-Q water as working solutions. Two microliters of working solutions was spiked in 18 µl of each cell extract and Milli-Q water for the fortified blank. Each sample was measured in triplicate at 1 µl injection volume. Both a non-weighted and a 1/c concentration weighted linear regression were fitted and matrix effects were calculated from slopes of regression lines.

## Results

### Transition selection and transition ion ratios for improved selectivity

**Fig. 1:**
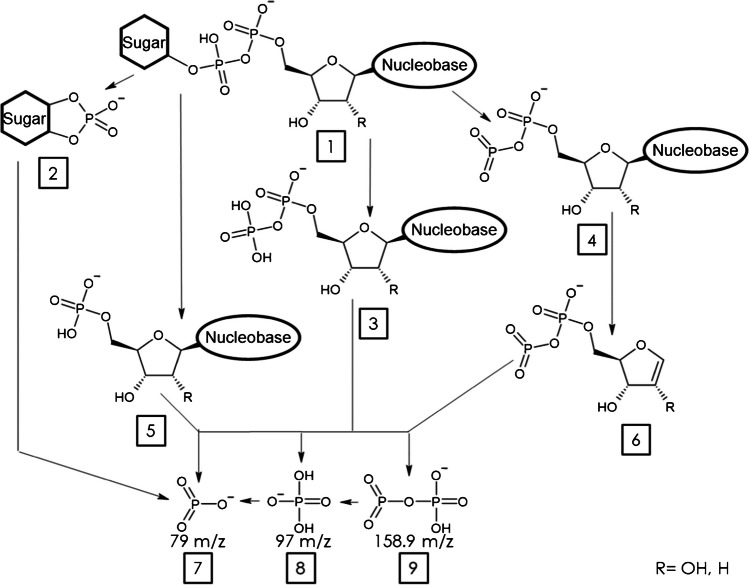
Generalized fragmentation scheme of nucleotide sugars in CID. 1, deprotonated nucleotide sugar [M-H]^−^; 2, fragment containing the sugar moiety bound to a phosphate anion; 3, nucleotide diphosphate anion fragment; 4, nucleotide dehydroxy-diphosphate fragment; 5, nucleotide monophosphate fragment; 6, dehydroxy-diphosphate ribose anion fragment; 7, metaphosphate anion/PO_3_^−^ fragment (79 m/z); 8, dihydrogen phosphate anion/PO_4_H_2_^−^ fragment (97 m/z); 9, dehydroxy-pyrophosphate anion fragment (158.9 m/z). R marks the hydroxyl group that is exchanged for hydrogen in dTDP-sugars

Most methods using MS/MS for detection of NS use no more than two transitions, focusing on the PO3^−^-phosphate ion (fragment 7, Fig. 1) for its high response and/or a more diagnostic product ion originating from the nucleotide [13, 14]. However, monitoring multiple transitions improves selectivity for (non-)isobaric nucleotide sugars as intensity ratios can differ between them. To establish our method, multiple transitions were derived from literature and a generalized fragmentation scheme was conceptualized (Fig. 1) [21, 22].

**Fig. 2:**
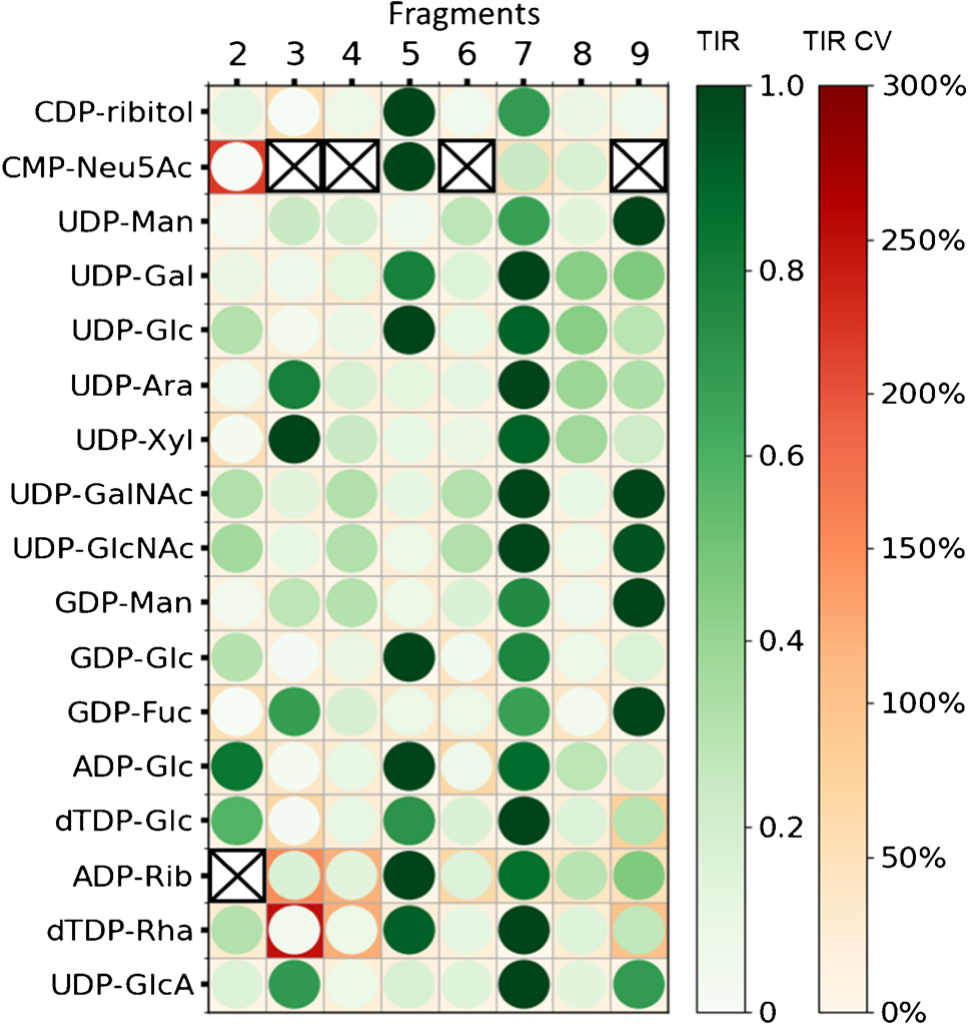
Transition ion ratio of all 17 nucleotide sugars included in the method (circles, normalized against maximum intensity transition). CV values for TIR are indicated by background coloring; highest intensity transition CV value is 0% due to the nature of normalization. Crossed out transitions are not monitored. Fragment numbers correspond to those used in Fig. 1. A facet grid bar graph ilustration for ease of data comprehension is shown in Supplementary Figure S2

By monitoring the fragments shown in Fig. 1 (fragments 2–9), all non-isobaric nucleotide sugars can be distinguished. Transitions and corresponding optimized collision energy (CE) values can be taken from Supplementary Table S1. In Fig. 2, we plotted the normalized transition ion ratios of all isobaric and non-isobaric NS to identify overlapping and discriminating patterns. For most compounds, fragment 7 (PO3^−^) is either the highest or second highest intensity product ion monitored, which is also used in most reported methods for the detection of nucleotide sugars [2, 13], as well as it being a terminal fragment as shown in Fig. 1. Several NS possess a distinct fragmentation spectrum when compared to their stereoisomers. For example, comparative analysis of the UDP-hexoses indicates a distinctive pattern for UDP-Mannose as compared to UDP-Glc and UDP-Gal with fragment 9 being more abundant and fragment 5 hardly detectable for UDP-Man. Similarly, for GDP-Man, fragment 9 was higher and fragment 5 was lower as compared to GDP-Glc. It has to be noted that ADP-Glc and GDP-Fuc are constitutional isomers but the TIR profile is specific for either one of these. CMP-Neu5Ac contains a phosphomonoester linkage, thus preventing the generation of fragments 3, 4, 6, and 9 that originate from phosphodiester linked NS (Figure S1 depicts the fragmentation scheme of CMP-Neu5Ac). The lack of ring formation by ribitol has no detected influence on the fragments monitored for CDP-ribitol. For adenosine-5′-diphosphate-d-ribose (ADP-Rib), fragment 2 was not included for monitoring, since ADP is bound to carbon 5 of the ribose sugar, thus not involving carbon 1, which is required to generate fragment 2. Although most of the NS possess a distinct fragmentation spectrum, the absence of diagnostic transitions in isobaric NS emphasises the need for chromatographic separation.

### Chromatographic separation using a weak anion-exchange/reversed-phase column

**Fig. 3:**
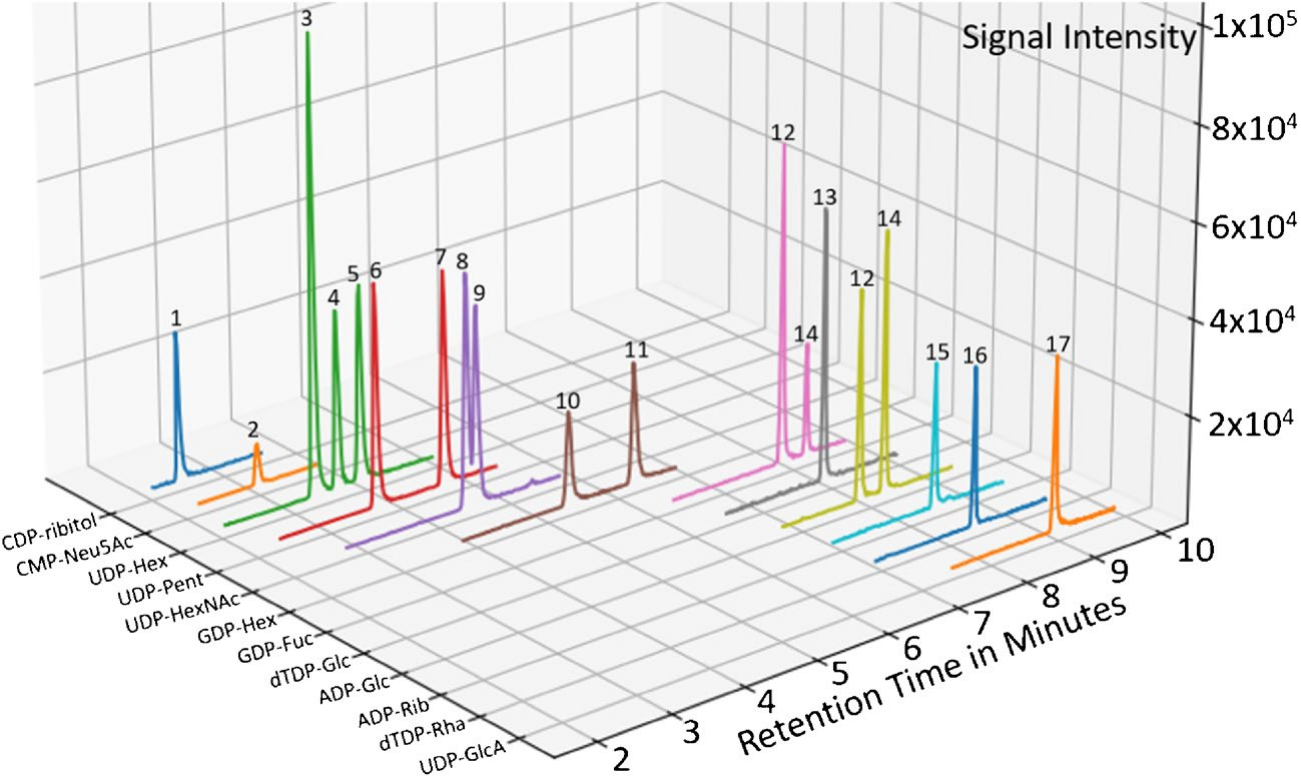
MRM chromatogram of a nucleotide standard mix with 200 nM concentration (CDP-ribitol was included from data of the control A cell line). All transitions are summed depending on precursor m/z. 1, CDP-ribitol; 2, CMP-Neu5Ac; 3, UDP-Man; 4, UDP-Gal; 5, UDP-Glc; 6, UDP-Ara; 7, UDP-Xyl; 8, UDP-GalNAc; 9, UDP-GlcNAc; 10, GDP-Man; 11, guanosine-5′-diphosphate-α-d-glucose (GDP-Glc); 12, GDP-Fuc; 13, 2′deoxy-thymidine-5′-diphosphate-α-d-glucose (dTDP-Glc); 14, ADP-Glc; 15, ADP-Rib; 16, dTDP-Rha; 17, uridine-5′-diphosphate-α-d-glucuronic acid (UDP-GlcA)

Considering the success of previous ion pairing methods, the combination of ionic interaction and hydrophobicity as retention mechanisms seemed promising. The phosphate groups of nucleotide sugars are negatively charged in the operational pH range of most chromatography methods, benefiting the potential of anion-exchange chromatography. We therefore opted for a mixed mode weak anion-exchange/reversed-phase column with the benefit of the ionic interactions retaining the nucleotide sugars as well as the hydrophobic interactions to take effect. Using ammonium acetate as counter ion, we obtained a nearly complete separation of isobaric nucleotide sugars. Our method showed similar retention order and characteristics as compared to established ion pairing separation methods that are believed to be governed though a combination of ionic and hydrophobic interactions. All three human UDP-hexoses, UDP-Glc, UDP-Gal, and UDP-Man, were baseline separated, as well as the GDP-hexoses and UDP-pentoses, while UDP-GalNAc and UDP-GlcNAc were partly separated. A representative MRM chromatogram of a nucleotide standard mix is shown in Fig. 3. Overall, the chromatographic separation was highly similar compared to the use of TBA as ion pairing agent [2] in a much shorter timeframe. Since cytidine and uridine are only 1 m/z apart, UDP-pentoses have to be separated from CDP-ribitol to eliminate interferences from natural ^13^C isotopologs and in-source dihydrogen losses from 1,4-elimination reactions in sugar moieties [22].

A general comparison of NS structures and the order of elution showed the charge state of the molecule to be of the strongest influence on the retention time. While all other UDP-sugars elute before 5 min, UDP-GlcA elutes last at around 9 min. It contains a carboxylic acid group in addition to the two phosphates also present in the UDP-sugars. Similarly, CMP-Neu5Ac which contains an additional carboxylic acid group elutes nearly as early as CDP-ribitol besides only containing one phosphate. Although the nucleobase seems to have an effect on the retention when comparing the nucleotide diphosphate glucoses (UDP-Glc < GDP-Glc < dTDP-Glc < ADP-Glc), the exact determination of their effect on retention is outside the scope of this paper.

### Method validation

**Fig. 4:**
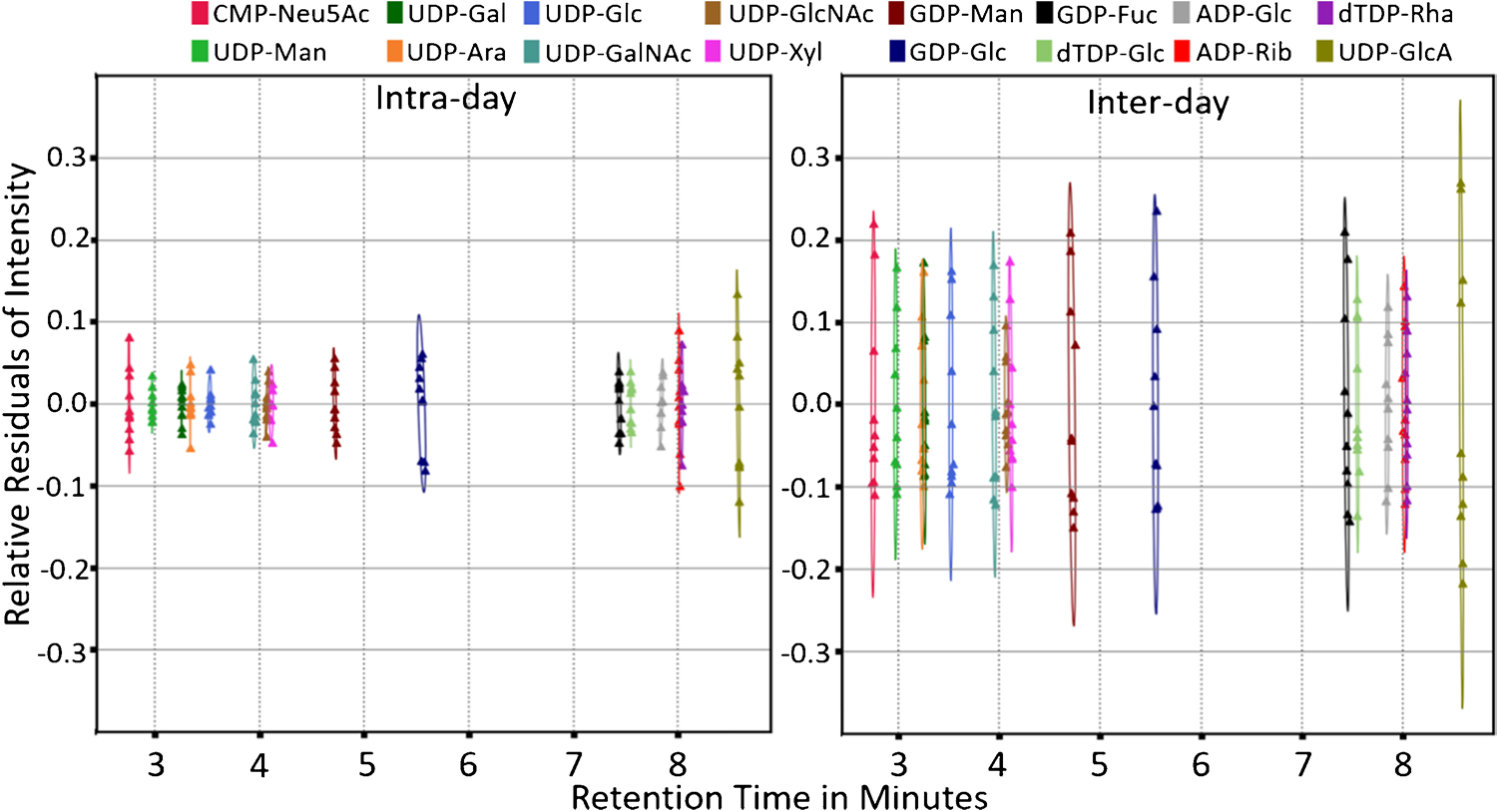
Intra-day and inter-day relative residuals of intensity plotted against the analyte retention time. Residuals of retention time were transformed to match compounds intra-day retention time. Confidence ellipses (*α* = 0.95) were plotted for both intra-day and inter-day values

As a next step to determine the applicability of the method for diagnostics of inherited metabolic diseases, a method validation was performed including instrumental LOD and LOQ, concentration-dependent LOD, analyte-dependent linear range, analyte carry-over and stability, intra- and inter-day variance, as well as matrix effect. For all NS, the preset declustering potential is well within the range of minimum in-source fragmentation. Leaving the optimum range of declustering potential leads to increased in-source fragmentation or loss of sensitivity from inadequate dissociation of adducts. Results for LOD, LOQ, and optimum declustering potential are presented in Supplementary Table S5. Determined linear ranges covered approximately three orders of magnitude (Supplementary Table S5) with good correlation between analyte concentration and peak area (*r* ≥ 0.98). The matrix effect of fibroblast extracts was assessed for ADP-glucose, GDP-glucose, and UDP-arabinose, not present in two control fibroblasts and was well within method variation (Supplementary Table S7). Intra-day variations did not exceed 10% CV for any nucleotide sugar; inter-day variation did not exceed 20% CV. Intra-day and inter-day standard deviation for the retention times is below 2 s for all nucleotide sugar standards (Fig. 4, Supplementary Table S6).

Sample stability was monitored across a total time span of 48 h, showing a variation within measurement CV% within 24 h, while a trend to decreased levels was observed after 48 h (see Supplementary Figure S3), indicating a preferred analysis interval within 24 h. The observed decrease in analyte peak area was between 1 and 12%. CMP-Neu5Ac is known to degrade through self-anhydride formation between the phosphate and carboxyl groups, leading to cleavage into CMP and Neu5Ac [23]. Still, under the tested conditions, no disproportionate degradation of CMP-Neu5Ac was detected.

### Analysis of nucleotide sugars in fibroblasts of patients with defects in sugar metabolism

**Fig. 5:**
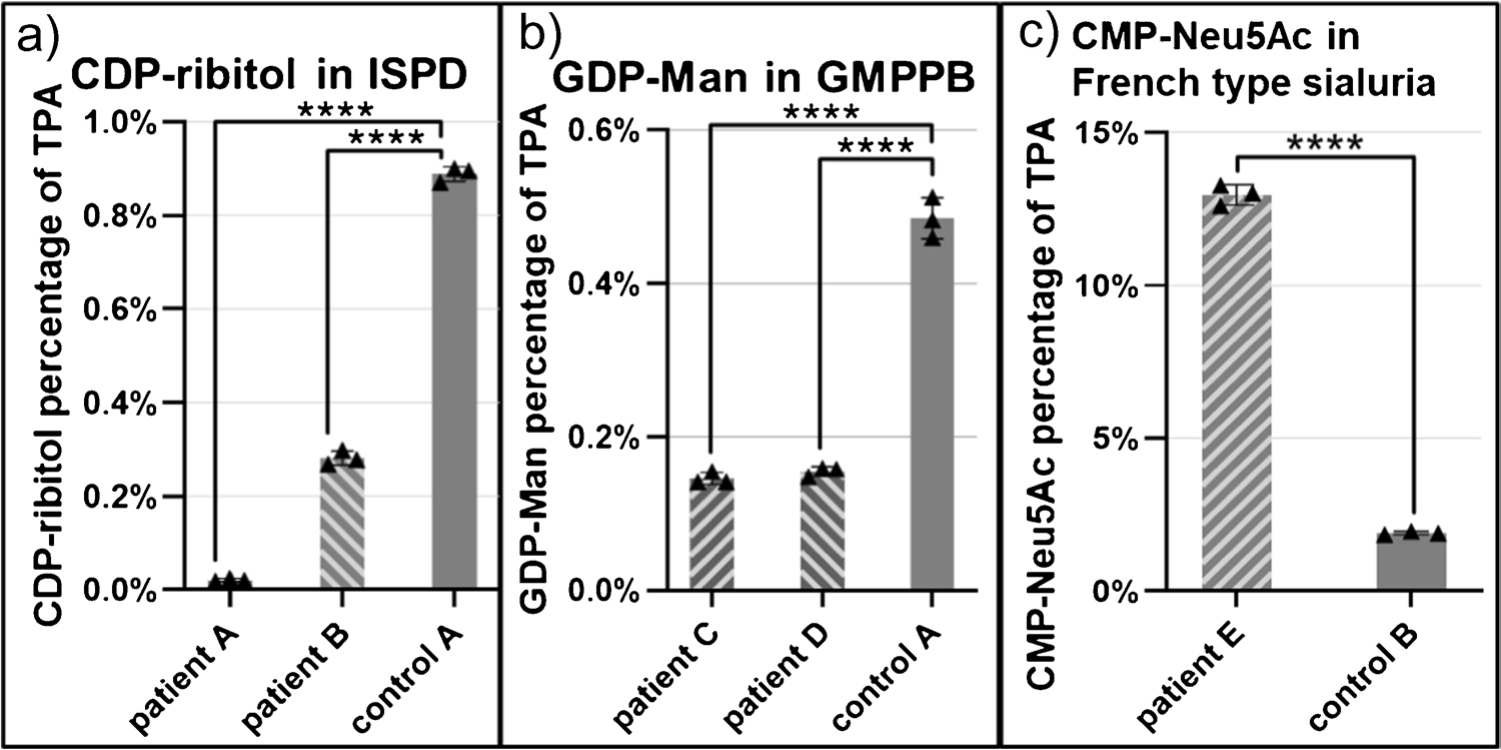
Changes in disease relevant nucleotide sugars normalized against total peak area of all nucleotide sugars. **a** The difference in CDP-ribitol level in CRPPA-deficient patients A and B compared to control cell line A. **b** The decrease in GDP-Man level in GMPPB-deficient patients C and D compared to control cell line A. **c** The increase in CMP-Neu5Ac level in French type sialuria patient E compared to control cell line B. Error bars represent the 95% confidence intervals. ****p < 0.0001 (adjusted *p*-value; one-way ANOVA). Full normalized nucleotide sugar profiles of patient and control cell lines can be found in Supplementary Figures S5 to S7

In order to examine the diagnostic capability of the presented methodology, we performed analysis of NS in fibroblasts of three different genetic defects. Two CRPPA-CDG fibroblast lines were tested, with a deficiency of CDP-ribitol pyrophosphorylase A (CRPPA; OMIM #616,052). CRPPA belongs to the family of isoprenoid synthase domain-containing proteins (ISPD) and activates ribitol-5-phosphate to cytidine-5′-diphosphate-l-ribitol (CDP-ribitol). CDP-ribitol is in turn used by the glycosyltransferases fukutin and fukutin-related protein for the synthesis of the tandem ribitol-phosphate motive of dystroglycan. Mutations in the *ISPD* gene can lead to loss of CRPPA activity resulting in dystroglycanopathy as the intracellular levels of CDP-ribitol are insufficient to maintain normal function of dystroglycan. The dysfunctional dystroglycan leads to limb-girdle muscular dystrophy [7]. In both patient lines, a highly significant decrease of CDP-ribitol was observed as compared to control (see Fig. 5a). While in the milder affected adult patient B, CDP-ribitol could still be detected at a decreased concentration, in the severely affected infantile patient A, CDP-ribitol could not be detected at all. This suggests a correlation of CDP-ribitol levels with phenotype severity.

In patients with defective mannose-1-phosphate guanylyltransferase beta (GMPPB; OMIM #615,320), the conversion of mannose-1-phosphate to its corresponding nucleotide sugar guanosine-5′-diphosphate-d-mannose (GDP-Man) is reduced, affecting mannosylation of proteins leading to a wide range of clinical phenotypes. This includes among others ataxia, cerebellar hypoplasia, limb-girdle muscular dystrophies (LGMD), liver diseases, seizures, and severe congenital muscular dystrophies (CMD) [6, 24]. Fibroblasts from GMPPB patients C and D showed a highly significant reduction in normalized GDP-Man levels (see Fig. 5b). There was no significant difference between both patient samples C and D (adjusted *p*-value > 0.9999).

French type sialuria (OMIM #269,921) is characterized by an increase in free *N*-acetylneuraminic acid (Neu5Ac) as a result of defective regulation of the sialic acid synthesis pathway. The rate-limiting reaction of this pathway is the synthesis of *N*-acetylmannosamine (ManNAc) from uridine-5′-diphosphate-*N*-acetylglucosamine (UDP-GlcNAc) catalyzed by the UDP-GlcNAc 2-epimerase domain of the GNE enzyme. ManNAc is then converted in a multi-step enzymatic cascade to CMP-Neu5Ac. Wild-type GNE possesses an allosteric binding site allowing feedback inhibition through CMP-Neu5Ac. In French type sialuria, a mutation in the allosteric binding site disables the feedback inhibition thereby increasing the concentration of both free Neu5Ac and CMP-Neu5Ac [8]. In fibroblasts of French type sialuria patients, CMP-Neu5Ac was strongly and significantly increased (> 500%, *α* = 0.05, *p* < 0.0001) depicting the expected accumulation due to dysregulated feedback inhibition (see Fig. 5c).

The ability to detect the disease-dependent alterations in NS level proves the diagnostic capability of our analytical method.

## Discussion

The aim of this paper was the development of an analytical method for separation and detection of nucleotide sugars without the need of ion-pairing buffers that in first instance is suitable for diagnostics of genetic glycosylation disorders in fibroblasts, and can be expanded to other research applications. The chromatographic separation presented in this paper is sufficient for separating nearly all of the 17 nucleotide sugars. By increasing the amount of monitored transitions, the ability to distinguish NS is improved as well. Method validation shows good reproducibility and a linear range sufficient for coverage of concentrations found in metabolite extracts generated with the used extraction protocol. Furthermore, the ability to detect differences between patient samples and healthy controls proves the diagnostic capabilities of our method and fulfilment of our initial aims.

Utilizing the weak anion-exchange/reversed-phase column forgoes problems associated with more common approaches like ion-pair reversed-phase or HILIC-based methods previously established. As ion-pair reagents contaminate instruments, particularly mass spectrometers, this reduces the flexibility of the instrument usage, often leading to dedicated ion-pair mass spectrometers. HILIC-MS on the other hand requires long equilibration times. If these equilibration times are to short, the reproducibility of the method can suffer greatly, making it practically useless for diagnostic purposes. Additionally, HILIC chromatography requires high amounts of organic solvents introducing additional cost for laboratory and environment. By using the mixed-phase weak anion-exchange/reversed-phase column, the separation of polar ionic nucleotide sugars could be achieved. On regular (fully end capped) reversed-phase columns, NS elute in the dead volume, as the hydrophobicity of NS is not sufficient for significant partition into the stationary phase. The introduction of the tertiary amino groups helps mitigating these problems, allowing the ion exchange mechanisms to take effect.

The method validation results are comparable to other targeted LC–MS/MS methods. The calculated LODs and LOQs are all below 10 nM although the applied method can lead to overestimation and might in some cases be lower than reported. However, the overestimation is beneficial as it improves analytical robustness and diagnostic value. The linear range and linearity values are sufficient for most diagnostic requirements, although repeated measurements and subsequent selection of optimal data sets might improve the consistency of the linear ranges across different NS. Intra-day variation in both analyte response and retention time is within the 10% coefficient of variation boundary; inter-day variation only shows UDP-GlcA to vary more than desired although still having a coefficient of variation below 20%.

For all three monitored CDGs, the changes in affected nucleotide sugar levels could be detected. In ISPD patient B, a significantly decreased CDP-ribitol level was detected. This implies low residual activity of the CRPPA enzyme. Meanwhile, in ISPD patient A, no CDP-ribitol was detected, suggesting a more severe clinical phenotype compared to patient B. These decreased levels of CDP-ribitol have already been reported by van Tol et al. in 2019 [7]. In both patients C and D affected by a deficiency in the GMPPB enzyme, the significantly decreased levels of GDP-Man were detected. This suggests either residual, but decreased activity of the GMPPB enzyme or limited synthesis by other pyrophosphatases. Lastly, the method was capable to detect the five-fold increase of CMP-Neu5Ac in the French type sialuria patient while remaining well within the method’s linear range. The results for these three genetic defects are promising for the future expansion of this method to other genetic defects in sugar metabolism that may affect NS levels. Examples include NANS-CDG, PGM3-CDG, and GFPT1-CDG for which currently no diagnostic methods are available. In view of the increasing evidence for the tissue- and cell-type-specific effects of such genetic diseases, the method may need to be expanded to other patient sample types such as blood cells. Further possibilities for use of the method include answering research questions regarding low abundance and uncommon NS that might provide further (disease specific) insight into human metabolism.

Altogether, weak anion-exchange/reversed-phase chromatography provides a facile non-ion pairing method for accurate and sensitive detection of nucleotide sugars in human-derived samples with the possible expansion to other organisms like plants or microorganisms or for the detection of synthetic nucleotide sugars.

### Supplementary Information

Below is the link to the electronic supplementary material.

Supplementary file1 (DOCX 1.98 MB)
