# Supplementary material for: Mixed-phase weak anion-exchange/reversed-phase LC–MS/MS for analysis of nucleotide sugars in human fibroblasts
Source: Anal Bioanal Chem. 2024 Apr 27;416(15):3595–604. doi: 10.1007/s00216-024-05313-w (PMC11156716; doi:10.1007/s00216-024-05313-w)

Supplementary Information

Mixed-phase weak anion-exchange reversed phase LC-MSMS for analysis of nucleotide sugars in human fibroblasts

Moritz Rahm^1^, Hanneke Kwast^2^, Hans Wessels­^2^, Marek Noga^3^, Dirk J. Lefeber^1,2^

Analytical and Bioanalytical Chemistry

This PDF file includes:

Table S1: Abbreviations of nucleotide sugars

Table S2: Transition m/z and corresponding CE values.

Table S3: Nucleotide sugar standard mix composition. x marking the concentrations stated in “Materials and Methods” section “Method validation”

Table S4: Analyte carry-over within linear range

Table S5: Blank noise-based intensity cutoff for LOD/LOQ, dilution series based LOD/LOQ, range of minimum fragmentation declustering potential and linear range results

Table S6: Intra- and Inter-day variation results

Table S7: Matrix effects of 3 representative nucleotide sugars in 2 fibroblast cell extracts

Figure S1: Fragmentation pathway adapted for CMP-Neu5Ac

Figure S2: Reconstructed MS/MS spectra of nucleotide sugars normalized to the highest intensity fragment ion.

Figure S3: Analyte stability over 48 hours in a standard mix

Figure S4: Complete normalized nucleotide sugar profile for two ISPD patients and one healthy control

Figure S5: Complete normalized nucleotide sugar profile for two GMPPB patients and one healthy control

Figure S6: Complete normalized nucleotide sugar profile for one French type sialuria patient and one healthy control

Table S1 gives an overview of all abbreviations of nucleotide sugars utilized in the paper.

Table S1: Abbreviations of nucleotide sugars.

| **Compound abbreviation** | **Compound** |
| --- | --- |
|  |  |
| **CDP-ribitol** | Cytidine-5'-diphosphate-L-ribitol |
| **CMP-Neu5Ac** | Cytidine-5'-monophosphate-*N*-acetyl-β-D-neuraminic acid |
| **UDP-Man** | Uridine-5'-diphosphate-α-D-mannose |
| **UDP-Gal** | Uridine-5'-diphosphate-α-D-galactose |
| **UDP-Glc** | Uridine-5'-diphosphate-α-D-glucose |
| **UDP-Ara** | Uridine-5'-diphosphate-β-L-arabinose |
| **UDP-Xyl** | Uridine-5'-diphosphate-α-D-xylose |
| **UDP-GalNAc** | Uridine-5'-diphosphate-*N*-acetyl-α-D-galactosamine |
| **UDP-GlcNAc** | Uridine-5'-diphosphate-*N*-acetyl-α-D-glucosamine |
| **GDP-Man** | Guanosine-5'-diphosphate-α-D-mannose |
| **GDP-Glc** | Guanosine-5'-diphosphate-α-D-glucose |
| **GDP-Fuc** | Guanosine-5'-diphosphate-β-L-fucose |
| **ADP-Glc** | Adenosine-5'-diphosphate-α-D-glucose |
| **dTDP-Glc** | 2'Deoxy-thymidine-5'-diphosphate-α-D-glucose |
| **ADP-Rib** | Adenosine-5'-diphosphate-D-ribose |
| **dTDP-Rha** | 2'Deoxy-thymidine-5'-diphosphate-rhamnose |
| **UDP-GlcA** | Uridine-5'-diphosphate-α-D-glucuronic acid |

Table S2: Transition m/z and corresponding CE values.

Numbering indicates the fragment denoted in fragmentation pathways shown in Figures 1 and S1. 1 is the precursor ion of the nucleotide sugar while 2-9 are the product ions after CID fragmentation. Collision energy (CE) values were optimized in accordance to conventional practice measuring intensities in a standard mix (see supplementary table S3 for composition).

| **Compounds** | | **CDP-ribitol** | **CMP-Neu5Ac** | **UDP-Hex** | **UDP-Pent** | **UDP-HexNAc** | **GDP-Hex** | **GDP-Fuc** | **ADP-Glc** | **dTDP-Glc** | **ADP-Rib** | **dTDP-Rha** | **UDP-GlcA** |
| --- | --- | --- | --- | --- | --- | --- | --- | --- | --- | --- | --- | --- | --- |
| **1** | **m/z** | 536 | 613.1 | 565 | 535 | 606.2 | 604.1 | 588.1 | 588.1 | 563.1 | 558.1 | 547.1 | 579 |
| **2** | **V** | -38 | -31 | -30.7 | -32 | -31.7 | -33.7 | -31 | -29 | -28 | - | -32 | -23 |
|  | **m/z** | 213 | 370.1 | 241 | 211 | 282 | 241 | 225 | 241 | 241 | 158.9 | 225 | 255 |
| **3** | **V** | -33 | - | -24 | -23 | -25.3 | -24.7 | -24 | -25 | -24 | -26 | -24 | -24 |
|  | **m/z** | 402 | 97 | 403 | 403 | 403 | 442 | 442 | 426 | 401 | 426 | 401 | 403 |
| **4** | **V** | -37 | - | -26 | -25.5 | -27 | -28.3 | -26 | -26 | -26 | -27 | -24 | -28 |
|  | **m/z** | 384 | 79 | 385 | 385 | 385 | 424 | 424 | 408 | 383 | 408 | 383 | 385 |
| **5** | **V** | -34 | -22 | -22 | -21 | -27.7 | -24 | -22 | -23 | -21 | -23 | -18 | -21 |
|  | **m/z** | 322 | 322 | 323 | 323 | 323 | 362.1 | 362.1 | 346.1 | 321 | 346.1 | 321 | 323 |
| **6** | **V** | -46 | - | -36 | -34.5 | -36.3 | -39.3 | -36.5 | -36.5 | -38 | -44 | -37 | -41 |
|  | **m/z** | 273 |  | 273 | 273 | 273 | 273 | 273^4^ | 273^4^ | 257 | 273 | 257 | 273 |
| **7** | **V** | -101 | -60 | -61.3 | -65 | -72.7 | -63.3 | -60 | -60 | -63 | -61 | -64 | -80 |
|  | **m/z** | 79 | 79 | 79 | 79 | 79 ^1^ | 79 ^1^ | 79^5^ | 79^5^ | 79 | 79 | 79 | 79 |
| **8** | **V** | -64 | -51 | -51 | -51 | -51 | -51 | -51 | -51 | -51 | -51 | -51 | -51 |
|  | **m/z** | 97 | 97 | 97 | 97 | 97 ^2^ | 97^2^ | 97^6^ | 97^6^ | 97 | 97 | 97 | 97 |
| **9** | **V** | -50 | - | -45.3 | -44 | -50.3 | -46 | -44.5 | -44.5 | -45 | -44 | -48 | -44 |
|  | **m/z** | 158.9 |  | 158.9 | 158.9 | 158.9^3^ | 158.9^3^ | 158.9^7^ | 158.9^7^ | 158.9 | 158.9 | 158.9 | 158.9 |

Table S3: Nucleotide sugar standard mix composition.

“Nucleotide sugar standard mix” is used as a reference run with each batch and was used to determine intra- and inter-day variation. Analyte stability standard mix was used to determine the on-board analyte stability at +4°C. Linearity standard mixes 1 to 4 were used to determine the linear range of the method. The concentrations are as follows: 10 nM, 33 nM, 100 nM, 330 nM, 1 µM, 3.3 µM, 10 µM, 33 µM. x marking the concentrations stated in “Materials and Methods” section “Method validation”

| **Compound** | **Concentration** | | | | | |
| --- | --- | --- | --- | --- | --- | --- |
|  | **Nucleotide sugar standard mix** | **Analyte stability standard mix** | **Linearity standard mix 1** | **Linearity standard mix 2** | **Linearity standard mix 3** | **Linearity standard mix 4** |
| **CMP-Neu5Ac** | 200 nM | 1 µM | x | - | - | - |
| **UDP-Man** | 200 nM | 1 µM | - | - | - | - |
| **UDP-Gal** | 200 nM | 1 µM | - | x | - | - |
| **UDP-Glc** | 200 nM | 1 µM | x | - | - | - |
| **UDP-Ara** | 200 nM | 1 µM | - | - | - | x |
| **UDP-Xyl** | 200 nM | 1 µM | - | - | x | - |
| **UDP-GalNAc** | 200 nM | 1 µM | - | - | - | x |
| **UDP-GlcNAc** | 200 nM | 1 µM | - | x | - | - |
| **GDP-Man** | 200 nM | 1 µM | - | x | - | - |
| **GDP-Glc** | 200 nM | 1 µM | - | - | - | x |
| **GDP-Fuc** | 200 nM | 1 µM | x | - | - | - |
| **ADP-Glc** | 200 nM | 1 µM | - | x | - | - |
| **dTDP-Glc** | 200 nM | 1 µM | - | - | x | - |
| **ADP-Rib** | 200 nM | 1 µM | x | - | - | - |
| **dTDP-Rha** | 200 nM | 1 µM | - | - | - | x |
| **UDP-GlcA** | 200 nM | 1 µM | - | - | x | - |

Table S4: Analyte carry-over within linear range.

The carry-over of nucleotide sugars was determined in accordance with the IUPAC convention. CDP-ribitol carry-carry over was not determined due to lack of a commercially available standard. As high level a concentration of 33 µM and 10 nM for the low level were chosen.

| Compound | Carry-over | Compound | Carry-over |
| --- | --- | --- | --- |
| CMP-Neu5Ac | 0.303% | UDP-Xyl | 0.011% |
| UDP-GalNAc | 0.006% | GDP-Glc | 0.039% |
| UDP-Ara | 0.008% | dTDP-Glc | 0.075% |
| GDP-Man | 0.020% | UDP-Glc | 0.007% |
| UDP-GlcNAc | 0.003% | dTDP-Rha | 0.007% |
| UDP-GlcA | 0.082% | ADP-Rib | 0.000% |
| UDP-Gal | 0.016% | ADP-Glc | 0.003% |
| GDP-Fuc | 0.015% |  |  |

Table S5: Blank noise based intensity cutoff for LOD/LOQ, dilution series based LOD/LOQ, range of minimum fragmentation declustering potential and linear range results. The minimum in-source fragmentation declustering potential range describes the applied voltage for which no decrease of analyte peak area greater than 5% could be determined. The linear range was determined for all nucleotide sugars with standards available at sufficient concentration with Pearson correlation of peak area and concentration.

| **Compound** | **Blank noise LOD cutoff (intensity /AU)** | **Blank noise LOQ cutoff (intensity /AU)** | **Dilution series based LOD (nM)** | **Dilution series based LOQ (nM)** | **Range of minimum fragmentation declustering potential (V)** | **Linear range (for 1 µL injection)** | **r** |
| --- | --- | --- | --- | --- | --- | --- | --- |
| **UDP-GlcA** | 248 | 652 | 1.1 | 5.6 | -35V - -100V | 10 nM - 10 µM | 0.98 |
| **ADP-Rib** | 344 | 882 | 3.0 | 6.8 | -25V - -115V | 10 nM - 10 µM | >0.99 |
| **dTDP-Rha** | 307 | 789 | 2.9 | 8.3 | -30V - -95V | 33 nM - 33 µM | >0.99 |
| **dTDP-Glc** | 515 | 1290 | 4.5 | 8.3 | -20V - -115V | 33 nM - 10 µM | >0.99 |
| **ADP-Glc** | 225 | 603 | 3.5 | 5.5 | -25V - -105V | 10 nM - 10 µM | 0.989 |
| **GDP-Fuc** | 196 | 512 | 2.2 | 4.0 | -15V - -115V | 10 nM - 33 µM | >0.99 |
| **GDP-Glc** | 102 | 265 | 0.8 | 2.7 | -25V - -120V | 10 nM - 10 µM | >0.99 |
| **GDP-Man** | 102 | 265 | 1.5 | 3.8 | -30V - -100V | 10 nM - 10 µM | >0.99 |
| **UDP-GlcNAc** | 301 | 855 | 1.5 | 5.5 | -35V - -95V | 10 nM - 3.3 µM | 0.988 |
| **UDP-GalNAc** | 301 | 855 | 2.9 | 6.4 | -35V - -95V | 10 nM - 10 µM | >0.99 |
| **UDP-Xyl** | 189 | 489 | 0.9 | 2.8 | -35V - -95V | 10 nM - 10 µM | >0.99 |
| **UDP-Ara** | 189 | 489 | 3.5 | 7.1 | -35V - -100V | 10 nM - 10 µM | >0.99 |
| **UDP-Glc** | 136 | 350 | 1.6 | 3.0 | -40V - -95V | 10 nM - 10 µM | >0.99 |
| **UDP-Gal** | 136 | 350 | 1.0 | 2.4 | -35V - -110V | 10 nM - 10 µM | >0.99 |
| **UDP-Man** | 136 | 350 | 0.4 | 0.9 | -35V - -110V | - | - |
| **CMP-Neu5Ac** | 61 | 153 | 1.1 | 3 | -30V - -75V | 10 nM - 10 µM | >0.99 |
| **CDP-ribitol** | 206 | 498 | - | - | - | - | - |

Table S6: Intra- and Inter-day variation results

Intra-day variation was determined from 10 consecutive measurements of a standard mix with 200 nM concentration of individual nucleotide sugars. Inter-day variation was determined from measurements of 10 standard mix aliquots with 200 nM concentration of individual nucleotide sugars on 10 consecutive days. The column was conditioned as described in the method section prior to the run. CDP-ribitol was excluded due to unavailability of a commercial standard.

| **Compound** | **Intra-day** | | | **Inter-day** | | |
| --- | --- | --- | --- | --- | --- | --- |
|  | **Average peak area** | **CV (%)** | **SD R_t_ (seconds)** | **Average peak area** | **CV (%)** | **SD R_t_ (seconds)** |
| **UDP-GlcA** | 114983 | 8.2 | 0.5 | 89850 | 18.6 | 2.0 |
| **ADP-Rib** | 93500 | 5.5 | 0.4 | 65310 | 8.1 | 1.8 |
| **dTDP-Rha** | 103840 | 3.8 | 0.1 | 56078 | 8.9 | 1.4 |
| **dTDP-Glc** | 182875 | 2.7 | 0.1 | 129656 | 9.0 | 1.9 |
| **ADP-Glc** | 174346 | 2.7 | 0.5 | 124215 | 7.9 | 1.5 |
| **GDP-Fuc** | 203052 | 3.1 | 0.5 | 147973 | 12.5 | 1.9 |
| **GDP-Glc** | 113477 | 5.4 | 1.2 | 88649 | 12.8 | 3.0 |
| **GDP-Man** | 377905 | 1.7 | 0.5 | 79245 | 13.6 | 2.6 |
| **UDP-GlcNAc** | 169723 | 2.3 | 0.5 | 139683 | 5.3 | 2.8 |
| **UDP-GalNAc** | 196286 | 2.7 | 0.5 | 162375 | 10.5 | 2.6 |
| **UDP-Xyl** | 221271 | 2.4 | 0.6 | 168547 | 9.1 | 2.2 |
| **UDP-Ara** | 207316 | 2.9 | 0.0 | 165746 | 8.8 | 2.4 |
| **UDP-Glc** | 187022 | 1.8 | 0.5 | 134278 | 10.8 | 2.3 |
| **UDP-Gal** | 162424 | 2.0 | 0.0 | 122444 | 8.4 | 2.3 |
| **UDP-Man** | 378783 | 1.8 | 0.0 | 294917 | 9.6 | 2.0 |
| **CMP-Neu5Ac** | 39701 | 4.3 | 0.2 | 17628 | 11.7 | 2.4 |
| **CDP-ribitol** | - | - | - | - | - | - |

Table S7: Matrix effects of 3 representative nucleotide sugars in 2 fibroblast cell extracts

| **Non-Weighted linear regression** | **Fortified blank** | | **Cell extract A** | | | **Cell extract B** | | |
| --- | --- | --- | --- | --- | --- | --- | --- | --- |
|  | **Slope** | **r²** | **Slope** | **r²** | **Matrix Effect (%)** | **Slope** | **r²** | **Matrix Effect (%)** |
| **UDP-Ara** | 689 | 0.997 | 678 | 0.986 | -1.6% | 639 | 0.996 | -7.3% |
| **GDP-Glc** | 415 | 0.997 | 429 | 0.985 | 3.4% | 405 | 0.996 | 2.4% |
| **ADP-Glc** | 551 | 0.996 | 562 | 0.983 | 2.0% | 527 | 0.995 | -4.4% |
| **Average** | - | | | | 1.26% | - | | -3.10% |
| **Weighted linear regression** | **Fortified blank** | | **Cell extract A** | | | **Cell extract B** | | |
|  | **Slope** | **r²** | **Slope** | **r²** | **Matrix Effect (%)** | **Slope** | **r²** | **Matrix Effect (%)** |
| **UDP-Ara** | 709 | 0.996 | 714 | 0.983 | 0.7% | 680 | 0.992 | -4.1% |
| **GDP-Glc** | 416 | 0.998 | 453 | 0.982 | 8.9% | 433 | 0.991 | 4.1% |
| **ADP-Glc** | 566 | 0.995 | 594 | 0.980 | 4.9% | 565 | 0.990 | -0.2% |
| **Average** | - | | | | 4.83% | - | | -0.06% |

Figure S1: Fragmentation pathway adapted for CMP-Neu5Ac.

As it contains a nucleotide monophosphate fragments 3, 4, 6 and 9 as present for nucleotide diphosphate sugars cannot be formed. Fragment 2 was included for systematic reasons although it only occurs at high analyte concentrations. Nucleotide sugar independent fragment m/z values are shown in the figure.


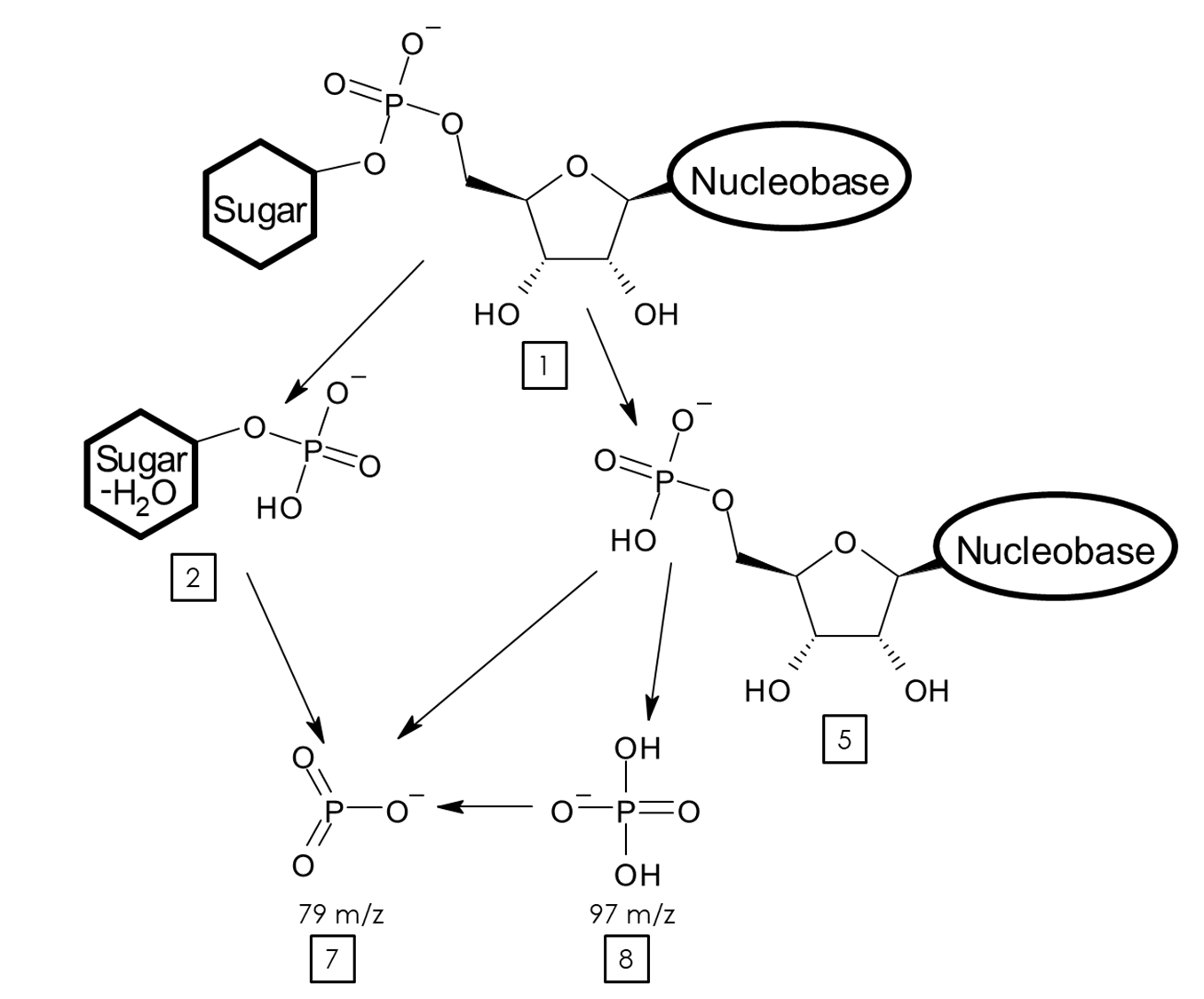


Figure S2: Reconstructed MS/MS spectra of nucleotide sugars normalized to the highest intensity fragment ion.

The reconstructed MS/MS spectra were generated from data for determining the linear range with the exception of UDP-Man and CDP-ribitol. UDP-Man transition ion ratio was extracted from Intra- and Inter-day variation experiments. CDP-ribitol was calculated from fibroblast data.


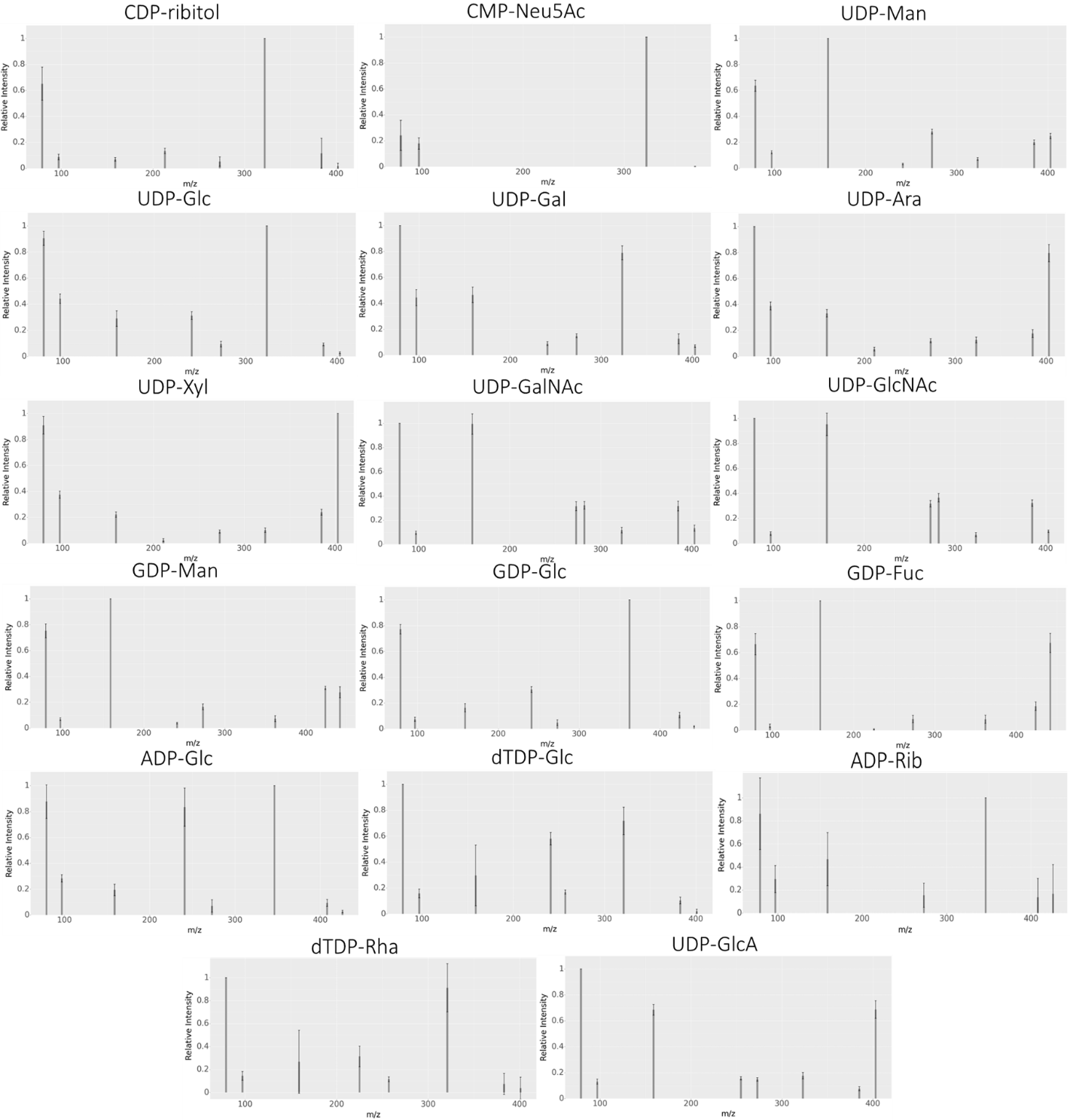


Figure S3: Analyte stability over 48 hours in a standard mix.

A 1 µM standard mix was left for 48 hours in the autosampler. The storage temperature was set to 4°C. Measurements were performed in triplicate at 0, 1, 4, 8, 12, 24 and 48 hours. The thick dashed line depicts the average change of all measured nucleotide sugars.


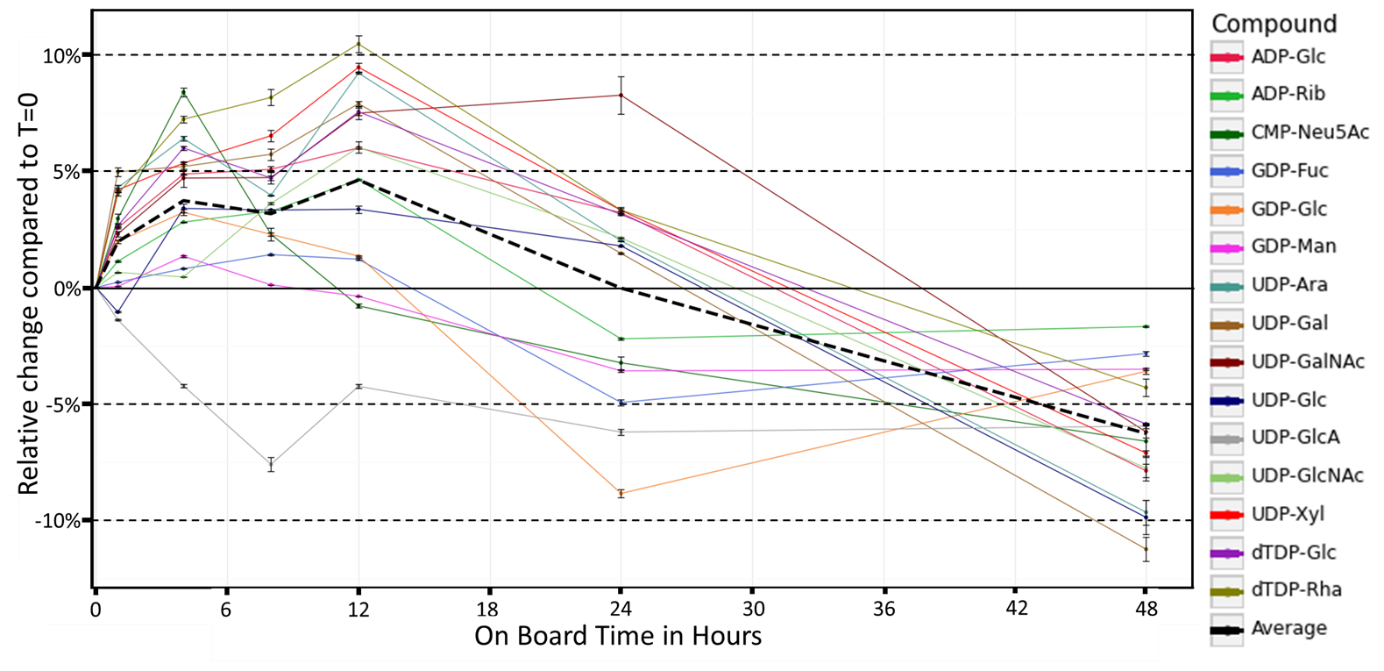


Figure S4: Complete normalized nucleotide sugar profile for two ISPD patients and one healthy control.

For improved visibility the y-axis was log_10_ scaled. Only minor changes can be seen for non-affected nucleotide sugars.


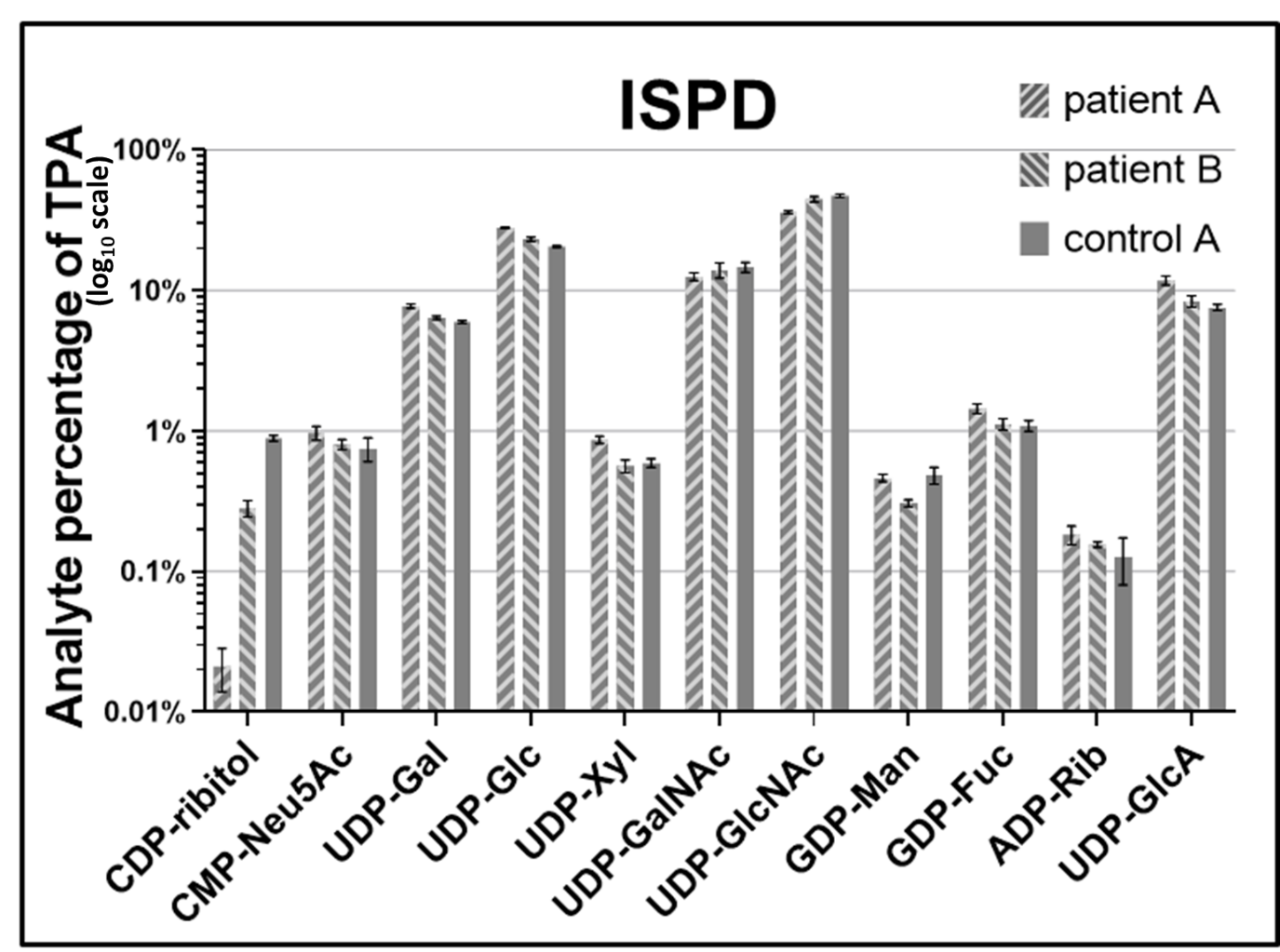


Figure S5: Complete normalized nucleotide sugar profile for two GMPPB patients and one healthy control.

For improved visibility the y-axis was log_10_ scaled. Only minor changes can be seen for non-affected nucleotide sugars.


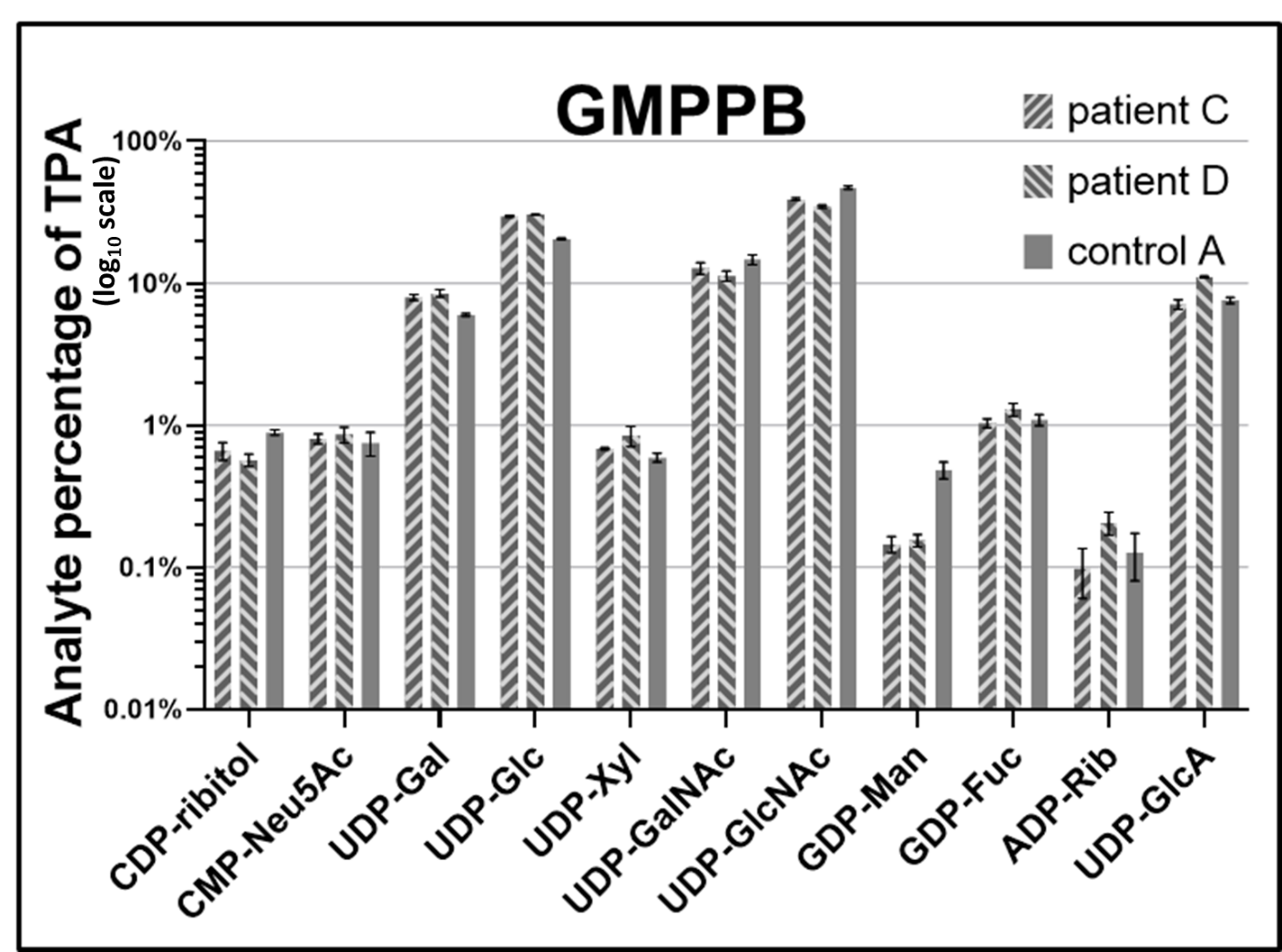


Figure S6: Complete normalized nucleotide sugar profile for one French type sialuria patient and one healthy control.

For improved visibility the y-axis was log_10_ scaled. Only minor changes can be seen for non-affected nucleotide sugars.


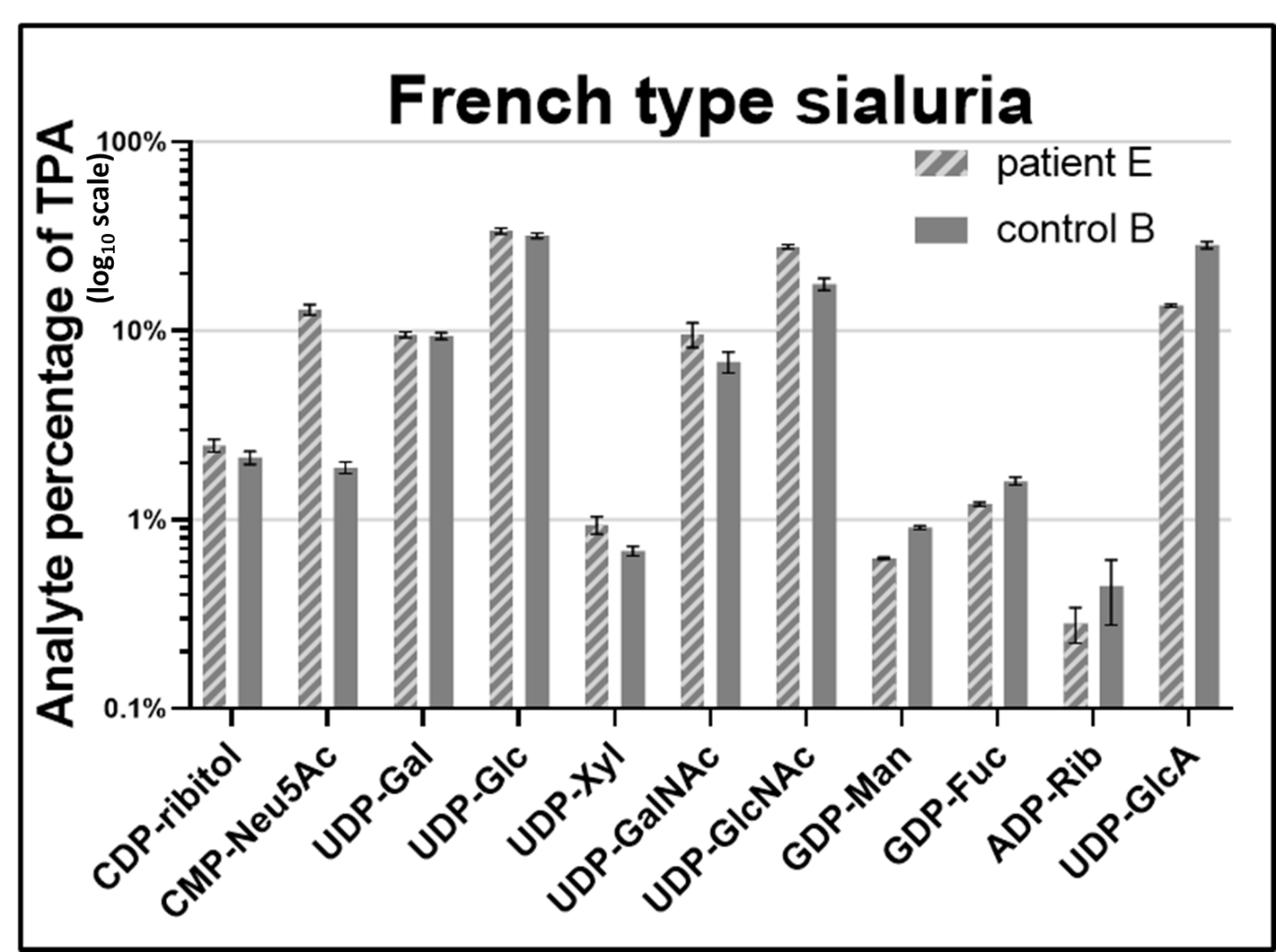

Supplement: Supplementary file 1 — Supplementary file1 (DOCX 1.98 MB) [file 216_2024_5313_MOESM1_ESM.docx]
